# Loxapine to control agitation during weaning from mechanical ventilation: a randomized controlled trial

**DOI:** 10.1186/cc14563

**Published:** 2015-03-16

**Authors:** S Gaudry, B Sztrymf, R Sonneville, B Megarbanne, C Clec'h, J Ricard, D Hajage, D Dreyfuss

**Affiliations:** 1 Hôpital Louis Mourier, Colombes, France; 2 Hôpital Béclère, Clamart, France; 3 Hôpital Bichat, Paris, France; 4 Hôpital Lariboisière, Paris, France; 5 Hôpital Avicennes, Bobigny, France

## Introduction

Weaning from prolonged mechanical ventilation (MV) in the ICU may be impeded by the occurrence of agitation. Loxapine had the ability to control agitation without affecting the efficacy of spontaneous ventilation in an observational study, justifying the implementation of a randomized controlled trial.

## Methods

We conducted a multicenter, placebo-controlled, parallel group, randomized trial at five French ICUs between November 2011 and November 2013. Patients (aged >18 years) under MV for more than 48 hours who were potential candidates for weaning from the ventilator and who exhibited agitation defined by a Richmond Agitation Sedation Scale (RASS) >2 after sedation withdrawal were randomly assigned to receive either loxapine or placebo. All participants were masked to group of allocation. After randomization, patients received 150 mg loxapine or placebo by nasogastric tube. RASS was monitored every 4 hours. A second dose of loxapine or placebo was administered if agitation persisted or worsened. In case of severe agitation, usual sedation (benzodiazepines and morphinic agents) was immediately resumed. Extubation was contemplated when patients were conscious and calm. The primary endpoint was the time between the first administration of loxapine or placebo and successful extubation (no reintubation in the following 48 hours). Three hundred patients were necessary to have 90% power to detect a 2-day reduction of weaning time in the loxapine group with a one-sided type I error rate of 5%.

## Results

The trial was discontinued after 101 patients had been randomized because of insufficient enrollment. Fifteen patients withdrew consent, leaving 86 patients for analysis. Forty-seven patients were assigned to the loxapine group and 39 to the placebo group. Median time to successful extubation was 3.2 days in the loxapine group and 5 days in the placebo group (RR = 1.2, 95% CI = 0.75 to 1.88; *P *= 0.45). During the first 24 hours, sedation was more frequently resumed in the placebo group (44% vs. 17%, *P *= 0.01). One patient had a transient seizure in the loxapine group.

## Conclusion

These results are consistent with the hypothesis of 2 days reduction of the median weaning time in the loxapine group, but the difference was not statistically significant. Loxapine reduces the need for resuming sedation during weaning from MV. Given the quality of the data and methodology, these results may be useful in future meta-analyses.

